# Annotations

**Published:** 1901-03-02

**Authors:** 


### ANNOTATIONS.

### Cisterns.

AMONG the various demands recently made upon the London householder by the water companies one is so thoroughly retrograde, from a sanitary point of view, that we feel bound to draw serious attention to it, and that is the proposal to enforce the provision of cisterns. As years go by new pipes and taps and other fittings must from time to time be necessary, and one can quite understand that the question who is to pay for them is sure to be discussed with a certain amount of acrimony. This, however, is a matter of pocket. The demand for cisterns stands on quite another footing. However much we may grumble at the occasional impurities met with in London water, and however questionable may be some of the proceedings of some of the water companies in regard to the management of their filter beds, the fact remains that the water is at its best when it comes fresh from the main. Every hour it remains in a cistern, even of the best and cleanest kind, it deteriorates, and, as cisterns generally are kept, a merely momentary passage through such a polluted reservoir is destructive of its purity. In good houses the difficulty is got over by providing taps on the "rising main" by means of which water can be drawn for drinking purposes before it has reached that centre of contamination the cistern. But in the houses of the poor it is not to be expected that a double supply will be provided, and we have to face the fact that to enforce the provision of cisterns in working-class houses, means practically to condemn their inhabitants to drink cistern water. It was largely because of the admitted evils connected with cistern-stored water that the provision of a constant service was made compulsory, and we venture to say that if the companies had honestly done their duty in this matter, and had given a really constant service, the wasteful habits which they now complain of would never have come into existence. What they did in many instances, however, was to supply a constant *dribble* instead of a constant *supply*. Of course the people had to let this dribble run into cans and buckets, and equally of course it often ran to waste, but if a really constant supply had been provided, a supply which came, as it does in some towns, frothing and foaming in full stream at high pressure at any time both day and night whenever the tap is turned, nobody would have used buckets and tubs and this waste would not have taken place. The waste is the direct result of the dribbling supply, and this is the direct outcome of the fact that the mains are too small and the pumping power is insufficient to provide an honest constant supply, such as was intended by Parliament when constant service was made compulsory.

### Biopsies.

A WORD is wanted which shall express the opposite of "necropsy." "Necropsy" means an investigation of or a seeing into a dead body, and is a good substitute for the time-honoured but indefinite and unfinished phrase "post-mortem." But with modern advances in the surgical art we are finding that a great deal of our knowledge of pathology is derived, not from necropsies, but from examination of living bodies in the operating theatre, and we want some word to express that side of operative work which gives us this particular sort of knowledge. Surgically, an operation is treatment, but what is it pathologically? Mr. Hutchinson[1] answers by giving us the word "biopsy," which, as the opposite of necropsy, will do very well. He tells us how very large and conspicuous are the gains to pathological knowledge which have accrued from careful observation of morbid conditions during life made by surgeons in the course of their operative work. First comes all that we know in regard to appendicitis. Opportunities for post-mortem examination of such cases in their early stages never occur. Modern surgery, however, has cleared the matter up, and the diseased conditions of the appendix in all their stages are now matters of familiarity. Then take ovarian disease and other diseases of the broad ligaments and pelvis; how much of our pathological knowledge depends upon biopsies made in the course of surgical intervention! If then we turn to the stomach and liver and gall-bladder, we find that the surgical procedures which are now undertaken for the treatment of these organs give opportunities of seeing and touching the parts and of gaining information as to their condition at stages of the disease far earlier than would be likely ever to come before our eyes in the post-mortem room.

[1] Polyclinic, February.

### A South African War Story.

FOR fiction displaying the most vivid qualities of invention an ordinary American newspaper is hard to beat. The following incident of the war has escaped the notice of all our war correspondents, but it is told in the *New York Herald* as the experience of a doctor who had been in South Africa. We give it, as the surgeon is said to have told it to some acquaintances at a club:—"There's one incident, which occurred at Pretoria, which I had almost forgotten, but which, I think, might interest you. It was really the most remarkable operation I ever saw. In a skirmish an Irishman, whose regiment I forget, was riddled with dumdums. His name was Patrick O'Hara, and he was one of the best men in the corps. He died like a hero and was carried to the rear. I happened to see the body as it was carried past me, and remarked on the fine physique of the man. Not long afterwards I was in the thick of the fight, in which were engaged also a body of Highlanders. One of the men I knew, and called out a few cheery words to him as I passed by. 'Eh, dochtor,' he replied, 'but we'll pound the deils afore munelicht,' and he rushed on with a bravery which everyone knows the Scotch possess. I thought no more of him until I came back, when, to my sorrow, I found my old friend Angus MacTavish—for that was his name—on a stretcher, with his upper lip clean blown off by one of the guns of the enemy. He was a horrible sight, and I was more than concerned what to do for him. Suddenly a thought struck me, which I immediately carried into effect. I found the body of Patrick O'Hara, which was still warm, and, giving MacTavish an anæsthetic, I sliced the top lip off Patrick and stitched it under the nose of MacTavish. The operation was perfect, though you may think me conceited in saying so. A month or so afterwards I was in Pretoria, not having seen MacTavish since the operation. One day I came across him and was delighted to see him looking so well. Evidently he was quite convalescent. I stopped him and said, 'Well, Angus, how goes it, my man?' To my astonishment he replied, in the richest brogue, 'Begorra, dochthor, I'm as roight as I can be, and faylin' illigant.' You see, the fact of the lip being transplanted had transferred the accent also." This is the story. How we wish the name of the surgeon had been given!

